# Medical Diseases of Infancy and Childhood

**Published:** 1899-02

**Authors:** 


Medical Diseases of Infancy and Childhood. By Dawson Williams, M. D., Physician to the East London Hospital for Children. In one 12mo volume of 629 pages, with eighteen illustrations. Philadelphia and New York: Lea Brothers & Co., Publishers. 1898.

The author presents in this volume a work on the diseases of children which, with the exception of its treatment of one subject, is very satisfactory. The book does not aim to be a complete treatise on diseases of children, but rather a manual of the medical diseases of childhood. The subject of pathology is but briefly discussed. In describing disease, the author draws a clear and accurate clinical picture, and it is for this clearness and accuracy in acquainting them with the various disorders of childhood that the young practitioners, for whom the book is chiefly intended, will especially value the work. The subject of treatment is well handled, being rational and modern and avoiding the inclusion of long lists of worthless remedies.

The exception to the general excellence of the work is found in the chapter devoted to infant feeding, which is altogether unsatisfactory. The only formula for modified milk given is the Meig's mixture, and this is found not in this chapter but in the appendix.

